# Phase Angle and Bio-Impedance Values during the First Year after Delivery in Women with Previous Excessive Gestational Weight Gain: Innovative Data from the Belgian INTER-ACT Study

**DOI:** 10.3390/ijerph18147482

**Published:** 2021-07-13

**Authors:** Margriet Bijlholt, Kate Maslin, Lieveke Ameye, Jill Shawe, Annick Bogaerts, Roland Devlieger

**Affiliations:** 1 Centre for Research and Innovation in Care (CRIC), Faculty of Medicine and Health Sciences, University of Antwerp, 2610 Antwerp, Belgium; margriet.bijlholt@uantwerpen.be (M.B.); Annick.Bogaerts@kuleuven.be (A.B.); 2 Research Unit Woman and Child, Department of Development and Regeneration, KU Leuven, 3000 Leuven, Belgium; lieveke.ameye@kuleuven.be (L.A.); jill.shawe@plymouth.ac.uk (J.S.); 3 Faculty of Health, University of Plymouth, Devon PL4 8AA, UK; kate.maslin@plymouth.ac.uk; 4 Royal Cornwall Hospital, Treliske, Cornwall TR1 3HD, UK; 5 Department of Obstetrics and Gynecology, University Hospital Leuven, 3000 Leuven, Belgium

**Keywords:** postpartum, body composition, gestational weight gain, pregnancy, bioelectrical impedance analysis

## Abstract

Phase angle (PhA) is a body composition parameter that measures changes in the amount and quality of soft tissue. Few studies have explored PhA in pregnancy or postpartum. The aim of this study was to explore the PhA during the first year postpartum in a Belgian cohort using data from the control group of the INTER-ACT study, an intervention trial targeting those with excess gestational weight gain. A secondary aim was to examine associations between PhA and potential explanatory variables. Women aged ≥18 with excessive weight gain in a singleton pregnancy and without a chronic disease were eligible. Data collection included anthropometry as well as demographic and lifestyle questionnaires at 6 weeks, 6 months and 12 months postpartum. Body composition, including PhA, was measured with the Tanita MC780SMA device. Data was analysed using correlation and mixed model analyses. A total of 509 participants (median age 31.2) were included. The median PhA at 6 weeks postpartum was 5.8°. Higher PhA values were seen in multiparous women (*p* = 0.02) but there was no association with any other lifestyle or demographic factors. PhA values were positively associated with muscle mass and BMI (r = 0.13, *p* = 0.004 and r = 0.18, *p* < 0.001) at 6 weeks postpartum. PhA increased slightly in the 12 months postpartum, which was related to a decrease in fat percentage (*p* = 0.004). Further research in the pregnant/postpartum population is needed to elucidate any links with perinatal or future health outcomes.

## 1. Introduction

During pregnancy and postpartum, the female body undergoes significant changes in order to support fetal and infant growth, development and lactation. These changes in lean mass, fat mass and weight are characterised by growth and increases in placental tissue, the uterus, blood volume and amniotic fluid, alongside changes in maternal body fat, breast tissue and total body water [1]. Recommendations for appropriate gestational weight gain (GWG), established by the National Academy of Medicine in 2009, are based on prepregnancy body mass index (BMI), with those who start pregnancy in the overweight or obese categories being advised to gain less weight than those in the healthy weight or underweight categories [2]. Both excess and inadequate GWG are linked to adverse outcomes, such as preterm birth, infant death and offspring weight status in childhood [3,4,5,6]. Excess GWG is also associated with postpartum weight retention persisting up to 15 years later [7]. Additionally, weight retention between the first and second pregnancy is associated with an increased risk for perinatal complications, with a greater risk observed in women who were of a healthy BMI in the first pregnancy [8,9,10].

Although the measurements of weight, height and BMI are simple and practical measurements to undertake in routine clinical practice, BMI does not distinguish between the proportions of lean mass, fat mass or total body water. There is therefore a valid argument for considering alternative measurements, particularly during pregnancy and postpartum. Although imaging methods are considered the most accurate for quantifying fat and lean mass, they are costly, not routinely available and/or are contraindicated due to radiation exposure [11]. In contrast, bioelectrical impedance analysis (BIA) is a low-cost, portable, non-invasive and practical body composition methodology for measuring total body water and body composition [12,13]. BIA is the degree to which a single or multiple currents are slowed (resistance) or stopped (reactance) as it passes through the body. The electrical conductivity of different biological tissues is determined by the amount of water they contain [12]. 

Phase angle (PhA) is a BIA parameter that was shown to be highly predictive of clinical prognosis and morbidity in a number of disease states [13,14]. PhA measures changes in the amount and quality of soft tissue mass [14]. It is an indicator of the distribution of body water between intra- and extracellular spaces, with higher values reflecting higher cellularity, membrane integrity and better cell function [13]. PhA is affected by age, gender and BMI, with considerable differences between reference values from different populations [13,14]. It is therefore recommended that individual results should be compared against population-specific reference values [1].

Looking specifically at the measurement of PhA in pregnancy or postpartum, only a few studies have sought to establish reference values [1] and/or explored changes in PhA over time in relation to other body composition variables [15,16,17]. These results are not externally generalisable as the studies have had small sample sizes [15,16] or described women who were predominantly underweight [17]. Therefore, the aim of this study was to describe and explore the evolution of PhA during the first year postpartum in a Belgian cohort of women who had excess GWG. A secondary aim was to examine associations between PhA, BMI and potential explanatory variables during the first year postpartum.

## 2. Materials and Methods

### 2.1. Recruitment

This study used data from the INTER-ACT study, an e-health-driven multicentre randomised controlled intervention trial targeting women who had excessive GWG [18,19]. Details of the INTER-ACT protocol and eligibility criteria were previously published [19]. In brief, women aged ≥ 18 years with a history of excessive GWG in a singleton pregnancy were recruited for the study at day 2 or 3 postpartum and randomised to the intervention or control arm of the study at 6 weeks postpartum. Recruitment took place between May 2017 and April 2019. For this analysis, data was only included for participants randomised to the control group.

Data collection included anthropometric and blood pressure measurements (see below for details), as well as demographic and lifestyle questionnaires, which were collected at 6 weeks, 6 months and 12 months postpartum [19]. Pregnancy data and medical history were also collected at recruitment. Pre-existing maternal diseases that possibly affect BIA, such as type I or II diabetes or renal disease, were exclusion criteria. 

### 2.2. Anthropometric Measurements

The anthropometric data consisted of maternal weight, height, waist and hip circumference and body composition measured using BIA. Maternal height was measured using a Seca—213 Leicester stadiometer (Seca GmbH & Co. KG., Hamburg, Germany). Maternal weight and body composition (fat mass, fat-free mass, muscle mass, total body water, extra-cellular water, intra-cellular water, organ fat and phase angle) were measured with the Tanita MC 780 SMA bio-electric impedance analysis device (Tanita Corporation, Tokyo, Japan). Waist and hip circumferences were measured with a Seca 201 (Seca GmbH & Co. KG., Hamburg, Germany) measuring tape in order to estimate the abdominal body fat. All measurements were performed according to the standard operating procedures to ensure data quality [19]. 

### 2.3. Measurement of BIA and Calculation of Phase Angle

A multi-frequency BIA device (Tanita MC780) (Tanita Corporation, Tokyo, Japan) was used in this study. This instrument applies alternating currents at 5, 50 and 250 kHz to measure resistance (R), reactance (Xc) and PhA. After entering details on gender, age and height, each participant stepped on the scale platform in light clothing and barefoot with feet on the four integrated electrodes. Grips with integrated electrodes were held in both hands and their arms were in a relaxed position alongside the body. Each measurement took approximately 20 s. 

The resulting measurements were automatically transferred to a computer, where they were interpreted by the software. Resistance (R) and reactance (Xc) were expressed as denominators in relation to height-squared [15,17]. Impedance (Z) and phase angle (PhA) were calculated using the formulae: Z^2^ = R^2^ + Xc^2^ and PhA = arctan(Xc/R) for each time point. 

### 2.4. Questionnaire Data

Two weeks in advance of every measurement appointment, participants received a personalised link to an online questionnaire. Sociodemographic information was only collected in the first questionnaire. A food frequency questionnaire (FFQ) comprising 48 food groups was included and used to calculate daily energy intake in kilocalories [20]. The International Physical Activity Questionnaire (IPAQ) was used to collect data on physical activity expressed as metabolic equivalent of task (MET) minutes per week and sedentary time in minutes per day [21].

### 2.5. Data Analysis

Data were analysed using SPSS version 27 (IBM Corporation, Armonk, New York, USA) and SAS version 9.4 (SAS Institute, Cary, North Carolina, USA). Participants with a pre-pregnancy BMI in the underweight range were excluded from the current analyses due to a low number. Measurements taken during a new pregnancy were also excluded from the current analyses. Participant characteristics were presented as number and percentage for categorical variables and median and interquartile range for continuous variables. In order to assess the association at 6 weeks postpartum (baseline) between the phase angle versus socio-demographic, weight-related and lifestyle determinants, the Spearman correlation was used for the continuous variables and the Wilcoxon test was used for categorical variables. In order to visualise the evolution over time of the phase angle during the first year postpartum, we used a scatterplot with lowess smoothing. In order to assess the evolution over time of the phase angle during the first year postpartum, mixed model analyses with a random intercept, random slope and unstructured working correlation matrix were performed. 

## 3. Results

### 3.1. Participant Characteristics

**Figure 1:**
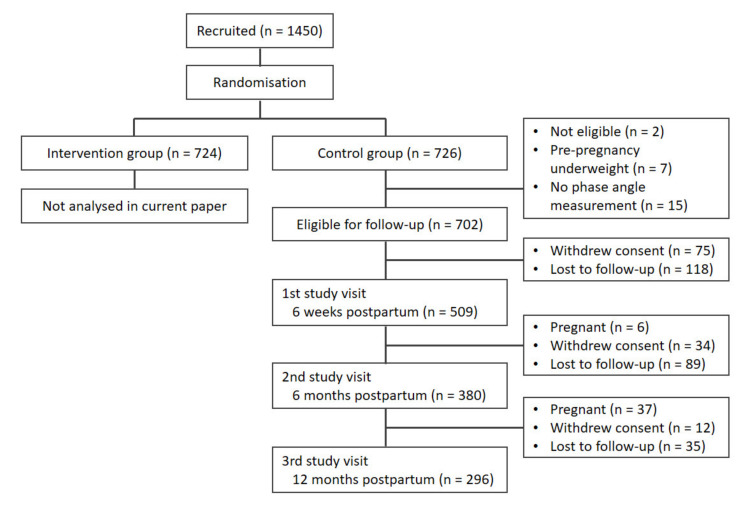
Flowchart of participant follow-ups.

A total of 509 participants were eligible for inclusion in the current analyses. Figure 1 shows the participant flowchart and Table 1 shows the baseline characteristics. PhA was not measured in 15 participants. The numbers of participants who withdrew consent and were lost to follow-up at each time point can be seen in Figure 1. Participants had a median age of 31 years, 56% were primiparous and 52% gave birth to a boy. The vast majority of participants were of white European descent (91%). 

### 3.2. Phase Angle Values at 6 Weeks Postpartum

Table 2 shows the association of the participants’ characteristics to the PhA at 6 weeks postpartum. Higher PhA values were associated with higher values of muscle mass and BMI: correlations of 0.13 (*p* = 0.004) and 0.18 (*p* < 0.001), respectively. Please note, muscle mass and BMI were highly correlated: correlation of 0.61 (*p* < 0.001). There was no statistical evidence for an association between PhA and fat percentage (*p* = 0.36), although there seems to be an association between PhA and waist–hip ratio: correlation of 0.16 (*p* < 0.001).

Higher PhA values were seen in multiparous women (*p* = 0.02). There was no association between PhA and any other lifestyle or demographic factor, including exclusive breastfeeding status.

### 3.3. Evolution in Phase Angle in the First Year Postpartum

**Figure 2:**
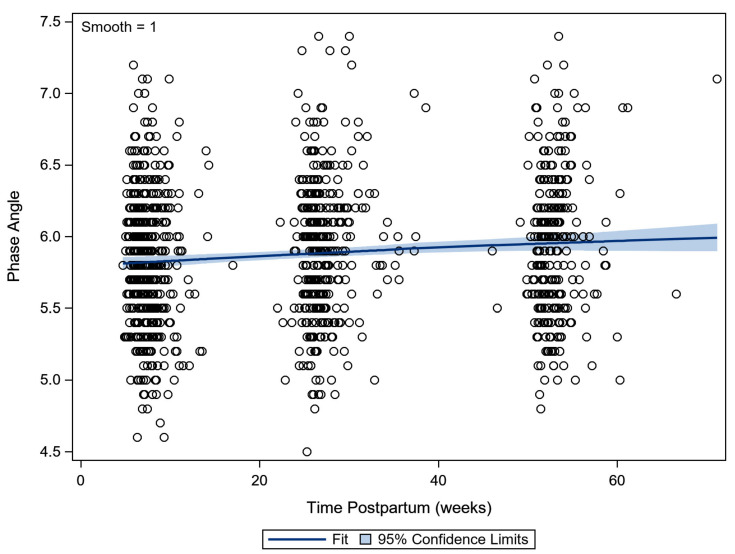
Scatterplot of the phase angle in the 1st year postpartum. A lowess smoother (with 95% confidence limits) indicated the evolution over time (lowess—locally weighted scatterplot smoothing).

The PhA increased slightly in the first year postpartum by 0.2° in total. The evolution was from a median of 5.8° at 6 weeks postpartum, to over 5.9° at 6 months postpartum, to 6.0° at 12 months postpartum (*p* < 0.001) (Figure 2).

The slight increase of PhA in the first year postpartum seems to be related to a decrease in fat percentage (*p* = 0.004), but no statistical evidence for an association to the evolution in muscle mass (*p* = 0.65) or waist-to-hip ratio (*p* = 0.77) was found.

## 4. Discussion

The assessment of changing body composition during pregnancy and lactation is challenging. However, due to the increasing prevalence of maternal obesity and potential obstetric complications, the ability to accurately measure and monitor body composition using simple, reliable and noninvasive methods during this phase is important. This study set out to describe such a method, focusing on the evolution of PhA during the first year postpartum in a Belgian cohort of women. A secondary aim was to examine the associations between PhA, BMI and fat mass percentage during this time. Using a cohort of >500 women who had excess GWG, at 6 weeks postpartum, PhA was related to muscle mass and BMI, but not to the fat percentage. There was, however, a slight increase in the PhA value during the first year postpartum, which was associated with a decrease in fat percentage. Higher PhA values were linked to higher cellularity, membrane integrity and better cell function.

The association between PhA and muscle mass was previously identified in both clinical and non-clinical populations and is a well-established association [14]. In a study of adults with obesity (60.8% female, mean age of 34.6) [22], PhA was positively correlated with lower- and upper-body maximal strength. Similarly, in malnourished or frail populations, it was found that PhA predicts muscle strength in women with anorexia nervosa [23] and is correlated with gait speed in older women [24]. Our finding that an increase in PhA correlates with a decrease in fat mass concurs with observations from weight loss studies. For example, Gerken et al. [25] identified that preoperative PhA predicts postoperative excess weight loss after bariatric surgery. Many variables, including surgery type, are thought to affect the outcomes of bariatric surgery [26]. Riberio’s [27] study in older women with obesity demonstrated decreases in fat mass, in conjunction with increased PhA, after 8 weeks of resistance training. These studies, however, did not address specific changes that occur in body composition that occur after pregnancy, or associated lifestyle factors, such as energy intake or sedentary activity.

Although the above-mentioned studies assessed the association between PhA and fat/muscle mass, few studies were undertaken using PhA in the postpartum population. In a Swedish study of 70 postpartum women, Ellegard et al. [16] reported that PhA was significantly higher at 15 months compared to 3 months postpartum, increasing from 6.33° to 6.44°, in accordance with a decrease in BMI. This concurs with the increase of 0.2° we observed between 6 weeks and 12 months postpartum. Although their sample was very similar to the present study, as measurements were undertaken in a European population of women as part of a lifestyle intervention, their PhA values were higher than we observed. This could be due to the smaller sample size or because their population only included women with overweight and obesity, whereas our inclusion criteria included healthy weight women, albeit with a history of excessive GWG. PhA may also be subject to impedance-analyzer-specific reference values, which depend on the equipment used [14]. The BIA analyzer used in their study underestimated fat mass whilst predicting implausible estimates of muscle mass compared to the double-labelled water method; therefore, their results are not comparable to ours.

After examining other studies of PhA during the postpartum timeframe, Shaikh et al. [17] reported on the evolution of PhA during pregnancy and until 12–18 weeks postpartum in a Bangladeshi population. The PhA in their sample was 6.1° in early pregnancy, decreasing to 5.7° late in pregnancy and increasing again to 5.9° at 12–18 weeks postpartum. Their study population of women was characterised by short stature, low body weight and experiencing their first pregnancy at a young age. Although the sample size was large, with 1141 measurements recorded postpartum, the mean BMI was 19.2 (SD 2) kg/m^2^, which is considered underweight by European standards. Overall, 46.1% of the Bangladeshi cohort were <20 years old at 12–18 weeks postpartum compared to our median age of 31.2 years at delivery. Of note, the authors detected age-related differences in BIA values, whereby PhA was generally lower among adolescents compared to older pregnant women, which is likely attributable to maturational differences of smaller body size and lower lean mass between younger and older women, as younger women grow towards peak stature and fat-free mass. The authors suggest the decrease in PhA in the third trimester, which was not observed in the better-nourished European pregnant populations [1,28], possibly indicates a more compromised body-cell mass profile at the outset of pregnancy and with advancing gestation. It also underlines the fact that BIA values are population-specific [29]. In a small USA-based study of only 15 women who were assessed during each trimester of pregnancy and at 8−10 weeks postpartum, Lukashi et al. [15] found that PhA did not change significantly during pregnancy and postpartum (staying stable at 6.6°). This was despite body weight and total body water (TBW) both progressively increasing during pregnancy and decreasing in the postpartum period, with the authors recommending that impedance vectors can be used to monitor changes in TBW during pregnancy and postpartum. The lack of change in PhA may have been due to the small sample size.

Among non-postpartum populations, Bosy-Westphal et al. [14] reported population reference values for PhA in a large German sample. Depending on BMI and age, the PhA in women of reproductive age ranged from 6.0° to 6.3°, which is slightly higher compared to the PhA values in our postpartum sample. Even higher values were reported among general American and Swiss populations [30,31]. It remains unclear whether the postpartum state of our population is the cause of these lower PhA values or whether these are normal differences observed between populations from different origins.

We investigated the association between PhA at 6 weeks postpartum and several lifestyle factors, namely, energy intake, sedentary activity, physical activity and breastfeeding status, finding no association. We are not aware of other research investigating associations between these parameters and PhA in the postpartum population. A study of Cameroonian women (*n* = 44) at 2–3 months postpartum found a lack of agreement between four BIA prediction equations. The authors surmised that changes in fat deposition and distribution during lactation or the higher hydration of FFM in lactating women could potentially modify the PhA measurement via the dispersion of the electrical current within the muscles. Previous research suggests that empirical prediction equations for body composition from BIA may no longer be valid in lactating women, recommending that further development and cross-validation of prediction equations from BIA that are specific to lactating women are needed [29].

At this stage, the precise clinical significance of PhA during the postpartum period is unknown; however, looking more broadly across the clinical nutrition literature, PhA was shown to be predictive of diverse clinical outcomes, including hydration and nutritional status [13,14]. Historically, the applicability of BIA to clinical practice has not been well understood or adopted [32], although in the first instance, establishing normative reference values is required, which we have sought to do in this analysis. The strengths of the study are the large sample size, measurements at multiple time points and robust statistical analyses that took lifestyle factors into account. The limitations are a potential lack of external generalisability due to the fact that only women with previous excess GWG were eligible to take part and the absence of a control group of women with adequate GWG.

## 5. Conclusions

In conclusion, we described and derived PhA values in a European cohort of postpartum women, demonstrating that PhA was associated with muscle mass and the increase in PhA during the first year postpartum was related to a decrease in fat percentage. Further research in the pregnant and postpartum population is needed to elucidate any links with perinatal or future health outcomes. Postpartum-specific variables, such as breastfeeding, should be taken into account and women with adequate GWG should be included.

## Figures and Tables

**Table 1:** Participant baseline characteristics.

		*n* = 509
Age at delivery, median (IQR)		31.2 (28.9–33.8)
Parity, *n* (%)	Primiparous	287 (56.4)
	Multiparous	222 (43.6)
Sex of infant, *n* (%)	Boy	266 (52.3)
	Girl	243 (47.7)
Education, *n* (%)	Up to 18 years of age	146 (29.4)
	Bachelor’s	194 (39.1)
	Master’s or higher	156 (31.5)
	Missing	13
Ethnicity, *n* (%)	White European	450 (90.7)
	Other ethnicity	46 (9.3)
	Missing	13
Pre-pregnancy BMI, *n* (%)	Healthy weight	251 (49.3)
	Overweight	179 (35.2)
	Obesity	79 (15.5)
Gestational weight gain in kg, median (IQR)	Overall	17 (15–20)
	Healthy weight pre-pregnancy	19 (17–21)
	Overweight pre-pregnancy	16 (13–19)
	Obese pre-pregnancy	13.4 (12–17)
PPWR at 6 weeks PP, median (IQR)	Overall	6.5 (3.9–9.0)
	Healthy weight pre-pregnancy	7.4 (5.2–9.6)
	Overweight pre-pregnancy	5.8 (3.2–8.4)
	Obese pre-pregnancy	4.4 (1.2–8.0)
BMI at 6 weeks PP, median (IQR)	Overall	27.7 (25.1–30.9)
Fat percentage at 6 weeks PP, median (IQR)	Overall	34.9 (31.9–38.6)
Muscle mass (in kg) at 6 weeks PP, median (IQR))	Overall	47.8 (44.8–51.0)
Waist–hip ratio at 6 weeks PP, median (IQR)	Overall	0.78 (0.75–0.82)
Phase angle (in degrees) at 6 weeks PP, median (IQR)	Overall	5.8 (5.5–6.1)
Energy intake (in kcal) at 6 weeks PP, median (IQR)	Overall	1387 (1132–1646)
MET-minutes per day at 6 weeks PP, median (IQR)	Overall	265 (127–487)
Sedentary time in minutes per day at 6 weeks PP, median (IQR)	Overall	300 (217–411)
Exclusive breastfeeding at 6 weeks PP, *n* (%)	Yes	274 (55.6)
	No	219 (44.4)
	Missing	16

BMI—body mass index; MET—metabolic equivalent of task; PP—postpartum; PPWR—postpartum weight retention.

**Table 2:** Association between phase angle and participant characteristics at 6 weeks postpartum.

		Association with Phase Angle	*p*-Value
Age (correlation)		0.03	0.45
Parity (median (IQR))	Nulliparous	5.8 (5.5–6.1)	0.02
	Multiparous	5.9 (5.6–6.2)	
Sex of infant (median (IQR))	Boy	5.8 (5.5–6.1)	0.40
	Girl	5.8 (5.5–6.2)	
Education (median (IQR))	Up to 18 years of age	5.8 (5.5–6.3)	0.46
	Bachelor’s degree	5.8 (5.5–6.1)	
	Master’s degree or higher	5.8 (5.5–6.1)	
Ethnicity (median (IQR))	White European	5.8 (5.5–6.1)	0.87
	Other ethnicity	5.8 (5.5–6.2)	
Body composition (correlation)	BMI	0.18	<0.001
	Fat percentage	−0.04	0.36
	Muscle mass	0.13	0.004
	Waist–hip ratio	0.16	<0.001
Lifestyle factors (correlation):	Energy intake (in kcal)	0.07	0.13
	MET-minutes/week	0.07	0.16
	Sedentary time/day	−0.08	0.12
Exclusive breastfeeding at 6 weeks (median (IQR))	No	5.8 (5.5–6.1)	0.85
	Yes	5.8 (5.5–6.2)	

BMI—body mass index; MET—metabolic equivalent of task.

## Data Availability

Data can be obtained from the corresponding author.

## References

[1] Berlit S., Tuschy B., Stojakowits M., Weiss C., Leweling H., Sutterlin M., Kehl S. (2013). Bioelectrical impedance analysis in pregnancy: Reference ranges. In Vivo.

[2] Rasmussen K.M., Yaktine A.L., Institute of Medicine, National Research Council (2009). Weight Gain during Pregnancy: Reexamining the Guidelines.

[3] Goldstein R.F., Abell S.K., Ranasinha S., Misso M., Boyle J.A., Black M.H., Li N., Hu G., Corrado F., Rode L. (2017). Association of Gestational Weight Gain With Maternal and Infant Outcomes: A Systematic Review and Meta-analysis. JAMA.

[4] Marchi J., Berg M., Dencker A., Olander E.K., Begley C. (2015). Risks associated with obesity in pregnancy, for the mother and baby: A systematic review of reviews. Obes. Rev..

[5] Bodnar L.M., Siminerio L.L., Himes K.P., Hutcheon J.A., Lash T.L., Parisi S.M., Abrams B. (2016). Maternal obesity and gestational weight gain are risk factors for infant death. Obes..

[6] Rogozinska E., Zamora J., Marlin N., Betran A.P., Astrup A., Bogaerts A., Cecatti J.G., Dodd J.M., Facchinetti F., Geiker N.R.W. (2019). Gestational weight gain outside the Institute of Medicine recommendations and adverse pregnancy outcomes: Analysis using individual participant data from randomised trials. BMC Pregnancy Childb..

[7] Nehring I., Schmoll S., Beyerlein A., Hauner H., von Kries R. (2011). Gestational weight gain and long-term postpartum weight retention: A meta-analysis. AM. J. Clin. Nutr..

[8] Teulings N., Masconi K.L., Ozanne S.E., Aiken C.E., Wood A.M. (2019). Effect of interpregnancy weight change on perinatal outcomes: Systematic review and meta-analysis. BMC Pregnancy Childb..

[9] Timmermans Y.E.G., van de Kant K.D.G., Oosterman E.O., Spaanderman M.E.A., Villamor-Martinez E., Kleijnen J., Vreugdenhil A.C.E. (2020). The impact of interpregnancy weight change on perinatal outcomes in women and their children: A systematic review and meta-analysis. Obes. Rev..

[10] Bogaerts A., Van den Bergh B.R., Ameye L., Witters I., Martens E., Timmerman D., Devlieger R. (2013). Interpregnancy weight change and risk for adverse perinatal outcome. Obstet. Gynecol..

[11] Marshall N.E., Murphy E.J., King J.C., Haas E.K., Lim J.Y., Wiedrick J., Thornburg K.L., Purnell J.Q. (2016). Comparison of multiple methods to measure maternal fat mass in late gestation. Am. J. Clin. Nutr..

[12] Most J., Marlatt K.L., Altazan A.D., Redman L.M. (2018). Advances in assessing body composition during pregnancy. Eur. J. Clin. Nutr..

[13] Norman K., Stobaus N., Pirlich M., Bosy-Westphal A. (2012). Bioelectrical phase angle and impedance vector analysis--clinical relevance and applicability of impedance parameters. Clin. Nutr..

[14] Bosy-Westphal A., Danielzik S., Dörhöfer R.P., Later W., Wiese S., Müller M.J. (2006). Phase angle from bioelectrical impedance analysis: Population reference values by age, sex, and body mass index. JPEN J. Parenter Enteral. Nutr..

[15] Lukaski H.C., Hall C.B., Siders W.A. (2007). Assessment of change in hydration in women during pregnancy and postpartum with bioelectrical impedance vectors. Nutr..

[16] Ellegard L., Bertz F., Winkvist A., Bosaeus I., Brekke H.K. (2016). Body composition in overweight and obese women postpartum: Bioimpedance methods validated by dual energy X-ray absorptiometry and doubly labeled water. Eur. J. Clin. Nutr..

[17] Shaikh S., Schulze K.J., Ali H., Labrique A.B., Shamim A.A., Rashid M., Mehra S., Christian P., West K.P. (2011). Bioelectrical impedance among rural Bangladeshi Women during pregnancy and in the postpartum period. J. Health Popul. Nutr..

[18] Bogaerts A., Bijlholt M., Mertens L., Braeken M., Jacobs B., Vandenberghe B., Ameye L., Devlieger R. (2020). Development and Field Evaluation of the INTER-ACT App, a Pregnancy and Interpregnancy Coaching App to Reduce Maternal Overweight and Obesity: Mixed Methods Design. JMIR Form Res..

[19] Bogaerts A., Ameye L., Bijlholt M., Amuli K., Heynickx D., Devlieger R. (2017). INTER-ACT: Prevention of pregnancy complications through an e-health driven interpregnancy lifestyle intervention—Study protocol of a multicentre randomised controlled trial. BMC Pregnancy Childb..

[20] Matthys C., Meulemans A., Van der Schueren B. (2015). Development and validation of general FFQ for use in clinical practice. Ann. Nutr. Metab..

[21] Craig C.L., Marshall A.L., Sjöström M., Bauman A.E., Booth M.L., Ainsworth B.E., Pratt M., Ekelund U., Yngve A., Sallis J.F. (2003). International physical activity questionnaire: 12-country reliability and validity. Med. Sci. Sports Exerc..

[22] Streb A.R., Hansen F., Gabiatti M.P., Tozetto W.R., Del Duca G.F. (2020). Phase angle associated with different indicators of health-related physical fitness in adults with obesity. Physiol. Behav..

[23] Popiołek-Kalisz J., Teter M., Kozak G., Powrózek T., Mlak R., Sobieszek G., Karakuła-Juchnowicz H., Małecka-Massalska T. (2020). Potential bioelectrical impedance analysis (BIA) parameters in prediction muscle strength in women with anorexia nervosa. World J. Biol. Psychia..

[24] Bittencourt D.C.D., Schieferdecker M.E.M., Macedo D.S., Biesek S., Silveira Gomes A.R., Rabito E.I. (2020). Phase Angle Reflects Loss of Functionality in Older Women. J. Nutr. Health Aging..

[25] Gerken A.L.H., Rohr-Kräutle K.K., Weiss C., Seyfried S., Reissfelder C., Vassilev G., Otto M. (2021). Handgrip Strength and Phase Angle Predict Outcome After Bariatric Surgery. Obes. Surg..

[26] D’Ettorre M., Bracaglia R., Gentileschi S., Tambasco D. (2013). Micro-and macroscopic tissue modifications after bariatric surgery: Effects of different procedures—A pilot study. Aesthetic Plast. Surg..

[27] Ribeiro A.S., Schoenfeld B.J., Dos Santos L., Nunes J.P., Tomeleri C.M., Cunha P.M., Sardinha L.B., Cyrino E.S. (2020). Resistance Training Improves a Cellular Health Parameter in Obese Older Women: A Randomized Controlled Trial. J. Strength Cond. Res..

[28] Piuri G., Ferrazzi E., Bulfoni C., Mastricci L., Di Martino D., Speciani A.F. (2017). Longitudinal changes and correlations of bioimpedance and anthropometric measurements in pregnancy: Simple possible bed-side tools to assess pregnancy evolution. J. Matern Fetal Neonatal Med..

[29] Medoua G.N., Nana E.S., Essa’a V.J., Ntsama P.M., Matchawe C., Rikong H.A., Essame Oyono J.L. (2011). Body composition of Cameroonian lactating women determined by anthropometry, bioelectrical impedance, and deuterium dilution. Nutrition.

[30] Barbosa-Silva M.C.G., Barros A.J., Wang J., Heymsfield S.B., Pierson R.N. (2005). Bioelectrical impedance analysis: Population reference values for phase angle by age and sex. Am. J. Clin. Nutr..

[31] Kyle U.G., Genton L., Slosman D.O., Pichard C. (2001). Fat-free and fat mass percentiles in 5225 healthy subjects aged 15 to 98 years. Nutrition.

[32] Elia M. (2013). Body composition by whole-body bioelectrical impedance and prediction of clinically relevant outcomes: Overvalued or underused?. Eur. J. Clin. Nutr..

